# Analysis of Neurosensory Changes in Orthognathic Surgery Using Saw or Piezoelectric Devices: A Scoping Review

**DOI:** 10.3390/jcm14103371

**Published:** 2025-05-12

**Authors:** Ailyn Navarrete, Víctor Ravelo, Leonardo Brito, Erick Vargas, Márcio de Moraes, Sergio Olate

**Affiliations:** 1 Grupo de Investigación de Pregrado en Odontología (GIPO), Facultad Ciencias de la Salud, Universidad Autónoma de Chile, Temuco 4780000, Chile; ailyn.nava@gmail.com (A.N.); l.brito.leal@gmail.com (L.B.); 2 PhD Program in Morphological Science, Universidad de La Frontera, Temuco 4780000, Chile; victor.ravelo.s@gmail.com; 3 Center for Research in Morphology and Surgery (CEMyQ), Universidad de La Frontera, Temuco 4780000, Chile; 4 Division of Oral and Maxillofacial Surgery, Hospital C.H.M., Chillan 3810525, Chile; evargas86@gmail.com; 5 Fellowship Orthognathic and Complimentary Facial Surgery, Universidad de La Frontera, Temuco 4811230, Chile; 6 Department of Oral Diagnosis, Division of Oral and Maxillofacial Surgery, School of Dentistry, State University of Campinas, Piracicaba 13414-903, Brazil

**Keywords:** piezoelectric, saw, orthognathic surgery, neurosensory alterations

## Abstract

Orthognathic surgery is a successful surgical method for correcting facial deformities, and the piezoelectric system can be used in place of or in addition to traditional tools like the reciprocating saw to perform osteotomies. This study assesses how using a reciprocating saw or a piezoelectric device exclusively affects neurosensory impairment. A review was performed following the PRISMA-ScR recommendations. A search was conducted until December 2024 in PubMed, Embase, and Web of Science. Clinical trials and quasi-experimental studies in English and Spanish were included. A total of nine articles were obtained for full-text review using inclusion and exclusion criteria. The selected articles included a total of 731 subjects. The age range of the studies included was between 18 and 49 years. Three of the six analyzed state that piezoelectric surgery positively correlates with neurosensory recovery, showing less surgical time and a less painful and faster postoperative recovery than the use of a saw. However, only two reported no significant statistical difference in sensitivity recovery between the saw and piezoelectric instruments. Despite the methodological heterogeneity among the studies included, the sample size, and the variability of factors, using a piezoelectric system typically shows a better postoperative recovery of sensitivity compared to using a saw.

## 1. Introduction

The introduction should Malocclusion affects approximately two-thirds of the world’s population [1] and is related to changes in facial structure with clinical problems, such as phonation and oral communication, difficulties in chewing, respiratory disorders, and others. Its etiology is usually multifactorial, influenced by genetic and environmental factors The classification of malocclusion is related to the position of some teeth [2] and can be present as the consequence of deficiencies in maxillomandibular position.

A poor three-dimensional relationship between the maxilla and mandible is frequently linked to malocclusions. In some clinical situations of moderate or severe discrepancies, orthognathic surgery is the treatment of choice, considered an effective surgical procedure that allows correction and restoration of muscular and masticatory function, phonation, respiration, and facial harmony [3].

In orthognathic surgery, the ultrasound system (piezoelectric) has become an alternative to conventional instruments like the saw. The use of piezoelectric (PE) reduces the risk of soft tissue injury and enhances less bleeding, which improves the accuracy of the osteotomy. Despite its advantages, the disadvantage has been raised that piezoelectric can take up 2 to 4 times longer than an osteotomy with a reciprocating saw and is more expensive than other surgical systems [4].

The different tools used in osteotomies can cause increased intraoperative bleeding, postoperative edema, and postoperative neurosensory alterations; the reciprocating saw commonly used in orthognathic surgery frequently poses challenges in control, limited visibility, and the conversion of electrical energy into a mechanical or rotary cutting motion, which can generate heat affecting adjacent structures like nerves and muscles, potentially leading to postoperative deterioration [5,6].

This study assesses differences in postoperative neurosensory impairment when using a piezoelectric device and a saw in orthognathic surgery.

## 2. Materials and Methods

### 2.1. Study Design

A literature review followed PRISMA-ScR [7] (Supplementary Materials Table S1) recommendations to answer the research question: Do neurosensory modifications differ following orthognathic surgery when an osteotomy uses piezoelectric versus saw systems? P: subjects who underwent orthognathic surgery; I: orthognathic surgery using piezoelectric surgery; C: orthognathic surgery using a saw; O: presence of neurosensory impairment and follow-up of three months or longer post-surgery.

### 2.2. Eligibility Criteria

We included studies evaluating the neurosensory response in orthognathic surgeries with piezoelectric and/or saw, in which a Le Fort osteotomy and/or sagittal mandibular ramus osteotomy was performed in a sample of more than 10 subjects with a follow-up of 3 months or more. Animal studies and studies in which the neurosensory response was evaluated in procedures other than orthognathic surgeries were excluded.

### 2.3. Source of Information and Search Strategy

A search was conducted from the year 2000 [8] (since it was in that year that the first article describing the use of piezoelectric in oral and maxillofacial surgery was published) to December 2024, which included articles in the PubMed, Embase, and Web of Science databases. Studies in English and Spanish were selected; there were no limitations in the type of study design. (((((((“Orthognathic Surgery”[Mesh]) OR (orthognathic surgeries)) AND (“Maxillary Osteotomy”[Mesh])) OR (osteotomy Lefort I)) AND (“Osteotomy, Sagittal Split Ramus”[Mesh])) OR (Sagittal split osteotomy)) AND (neurosensory disturbance)) OR (neurosensory alterations).

### 2.4. Study Selection and Data Extraction

The complete list of identified references was imported into the Mendeley 2.90.0 software (Reference Management, Elsevier, London, UK), where duplicates were automatically removed. Titles and abstracts were independently screened for eligibility by two investigators. In case of discrepancy, consensus was obtained by discussion or consultation with a third reviewer. References that appeared to fulfill the inclusion criteria were reviewed in full text by the same reviewers.

Data extraction was performed by two reviewers using a predefined and standardized data form:

(a) Study group data (number of patients, sex, age, and race);
(b) Research data (prospective or retrospective nature of the study, surgical procedure, surgical technique, and complementary technique);
(c) Type of data analyzed (clinical methods to determine the presence of paresthesia and follow-up).

## 3. Results

**Figure 1:**
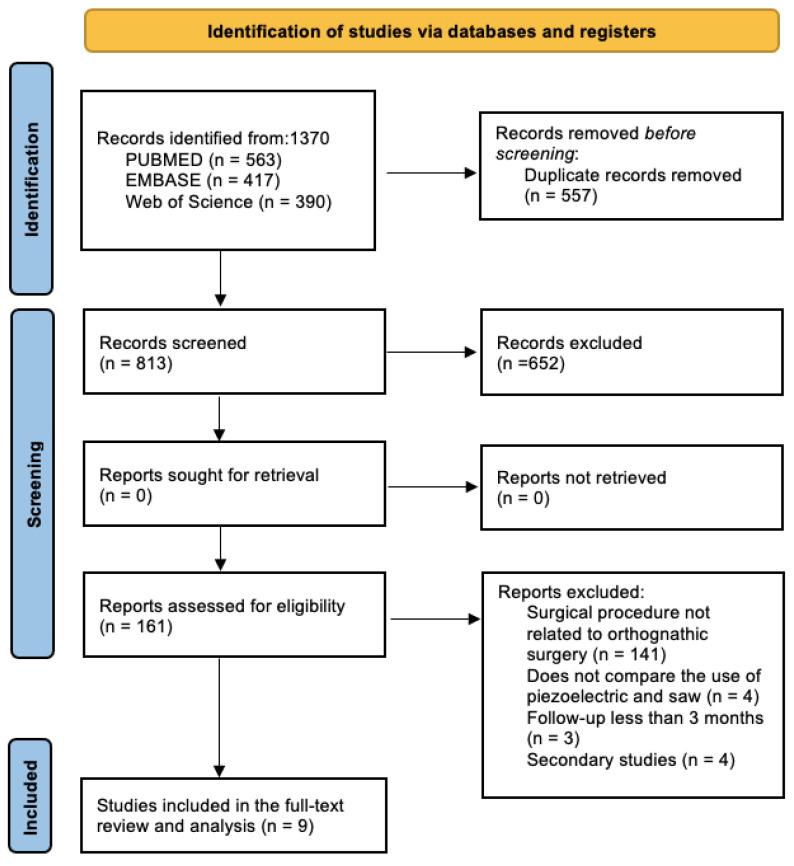
Flowchart of search and selection of included items.

The search conducted using the three metasearch engines yielded 1370 results, of which 557 were excluded due to duplication. A total of 813 articles were obtained for review based on title and abstract, using inclusion and exclusion criteria. Of all the studies analyzed, nine articles [9,10,11,12,13,14,15,16,17] were selected for full-text analysis (Figure 1).

The selected articles included a total of 731 subjects. The age range of the studies included was between 18 and 49 years. Concerning sex, 242 subjects were male (40%), and 371 were female (60%); only one study [8] did not mention the number of male and female patients. All the selected studies had a minimum follow-up of 3 months, ranging from 1 to 36 months (Table 1). Regarding the country where the study was conducted, only two mentioned the country where it was performed: Italy [11] and Brazil [16]. Four studies were prospective in design, and two were retrospective.

A total of 597 orthognathic surgeries were performed, of which 90 were performed in one hemiarch with piezoelectric (PE) and the other with a saw [12,13,15]. Two hundred thirty-seven operations were performed only with piezoelectric, 114 operations only with saw, and only one study did not mention how many surgeries were performed with piezoelectric or saw [14]. All the selected studies performed post-surgical follow-ups: one study performed follow-up up to 36 months [16], three studies up to 12 months [11,13,15], two studies up to 6 months [12,17], and one study up to 3 months [8,9,14]. Four studies assessed the measurement of neurosensory alterations using the light brush technique and two-point discrimination. In contrast, three studies evaluated it using the visual analog scale, neurosensory tests, light touch, and the two-point discrimination test [13,14,17]. Only one study was evaluated using the Semmes–Weinstein test [12].

Some authors reported that paresthesia after orthognathic surgery lasted up to 6 months [11,12,13,15,17], and that in the period between 2 and 3 years, the changes were insignificant. In the study by Da Costa et al. [16], of 376 patients, 152 presented paresthesia at 6 months. In the study by Cascino et al. [17], of a total of 100 patients, only 40 recovered skin sensitivity during the first month, while the study by Köhnke et al. [13] indicated that all patients presented postoperative paresthesia and that 50 presented paresthesia that gradually improved, reaching preoperative levels at 12 months. Sobol et al. [11] reported that in a study with 20 patients, none presented postoperative alterations in the sensitivity of the lingual nerve. Kokuryo et al. [14] reported that among 67 patients, only 22 presented paresthesia at 3 months, and Monnazzi et al. [12] stated that, of the 20 patients who underwent surgery, the side of the mandible operated on with piezoelectric recovered sensitivity, while the side treated with the saw took 6 months to recover sensitivity. Bertossi et al. [11] observed that at 12 months, all subjects operated on with piezoelectric instruments had recovered sensitivity within a mild to moderate range, whereas in the saw group, eight out of fifty-five subjects had not yet recovered sensation. Of those eight subjects evaluated at 12 months, only three had regained slight sensitivity in the area.

One study mentioned that sensitivity recovery was faster in patients operated on with piezoelectric, with a median recovery of 97 days, compared to the saw that presented a median recovery of 123 days [15]. In another study, however, it is mentioned that 16 patients out of 35 treated with piezoelectric and 28 out of 32 treated with saw did not recover sensitivity until the end of that study [14].

Regarding anatomy, the areas most affected were the lower lip and chin [12,16]. Of the six studies analyzed, three mentioned that surgeries performed with piezoelectric were positively associated with neurosensory recovery, in addition to a shorter operating time and a less painful and faster postoperative recovery compared to the saw, which presented a greater demand for analgesics and hospital stay [12,15,17].

In relation to Table 2, two studies mentioned that there was no significant difference in sensitivity recovery between the saw and the piezoelectric [12,14], and only one study included an analysis of risk factors for the development of sensory disturbances, which are as follows: advanced age, extensive mandibular advancement, and surgical procedures for nerve detachment. They also pointed out that in the analysis of the recovery of neurosensory alterations at 3 years, only 41 still presented some type of paresthesia without mentioning how many of these were performed with a saw or piezoelectric.

## 4. Discussion

The reciprocating saw has been a fundamental tool in osteotomies in orthognathic surgery for decades, achieving satisfactory results with low complication rates [18]. However, the introduction of the PE in the early 2000s marked a significant advance in technology for maxillofacial surgery [8]. Its characteristics, such as cooling, lower heat generation, and precision cutting that preserves soft tissues, make deep osteotomies possible and minimize surgical trauma, potentially reducing damage to adjacent neurovascular structures. This accuracy and reduced tissue damage appear to be a key advantage of PE [10].

The descriptive analysis of the six studies, which included 633 patients, showed no clinical differences between the two devices, and all studies reported the presence of postoperative paresthesia. However, differences were observed in the recovery time from these sensory alterations, with a shorter recovery time after six months in patients who underwent surgery with piezoelectric devices. This finding suggests that, although both methods may result in a similar incidence of paresthesia, piezoelectric surgery may be associated with faster sensory recovery. It is important to note that factors such as age, the type of surgery (whether advancement or setback), and the presence of sagittal facial abnormalities and/or asymmetries can influence the occurrence of facial sensory impairment.

The proximity of maxillomandibular osteotomies to sensitive nerve structures such as the inferior alveolar, infraorbital, and lingual nerves explains the high prevalence of postoperative neurosensory disturbances. Specifically, the sagittal ramus mandibular osteotomy (SRMO) shows an incidence of paresthesia ranging from 25% to 98% in the immediate postoperative period and from 10% to 30% in the long term [19]. This variability in the incidence of paresthesia could be influenced by several factors, including patient age, surgeon experience, type of instrumentation used, bone density, magnitude of surgical movement, concomitant genioplasty, and kind of fixation used [20,21].

Previous studies [22] have reported benefits of PE in neurosensory recovery, observing greater sensory function at 12 months in patients operated on with this technique compared to the saw. However, other studies like that by Köhnke et al. [13], which analyzed 50 patients, found no significant differences between the two methods. This discrepancy in findings could be attributed to the inherent variability in clinical studies, including differences in the characteristics of the study population, the surgical protocol used, and the surgeons’ experience. It has been reported that a less experienced surgeon is up to three times more likely to experience postoperative complications [23]. Although a high percentage of neurosensory disturbances resolve within the first 6 to 12 months, persistent disturbances may affect the patient’s quality of life and require additional interventions.

The development of the surgical technique also determines the neurosensory and postoperative recovery. For example, Raffaini et al. [24] used a hybrid use of the piezoelectric system in conjunction with chisels. In contrast, Olate et al. [25] used the PE tip exclusively without chisels or saws. It is possible to speculate that the postoperative outcomes could vary between the two scenarios. Although the piezoelectric system tends to increase the duration of the surgical procedure by approximately 30 to 50%, especially when cutting dense cortical bone, it has also been observed to reduce the incidence of sensitivity due to the preservation of soft tissues, including the perineurium of the nerve [26]. Several authors [27,28] mention that higher rotational speeds result in greater temperature ranges with smaller temperature variations and a reduction in surgical time, but that immediate cellular damage is associated with both the magnitude and duration of heat exposure. For their part, Delgado-Ruiz et al. [29], through in vitro studies, evaluated temperature variations in dense and trabecular bone tissue using two piezoelectric devices at 30 kHz, and they observed no differences in the temperature and time recorded during osteotomies performed with both devices. However, dense bone resulted in greater changes in temperature and time compared to osteotomies in trabecular bone.

Sagittal ramus mandibular osteotomy is one of the most common surgical procedures used to correct mandibular deformities such as prognathism, retrognathism, and facial asymmetry [30]. The anatomical conditions observed in dentofacial deformities could be associated with the position of the nerve and the internal mandibular canal [31]; in terms of skeletal class, individuals with Class III deformities present a higher chance of nerve paresthesia during mandibular setback than during mandibular advancement and then Class II patients. An anatomical option to explain these differences could be because Class III patients have a shorter distance between the buccal side of the cortical bone and the mandibular canal in the mandibular ramus than those with Class II skeletal deformities [32]. Those anatomical conditions are not included in the main articles to compare the treatment using PE or saw, and this bias in sample inclusion and selection can also explain the results in some papers published in this field.

Although the use of piezoelectric instruments during osteotomy allows for a precise cut on mineralized tissue and helps protect neurovascular structures by reducing thermal injury and microfractures [33,34], in the study by Gopinath et al. [35], an evaluation was carried out on the incidence of neurosensory disturbances following SRMO for mandibular setback. They observed that, among the 31 sites where the alveolar nerve was in the distal segment, there was no nerve manipulation, and the presence of postoperative neurosensory issues was low. In contrast, in the 14 sites where the nerve was found in the proximal segment, it had to be released in 60% of the cases, which presented a higher frequency of neurosensory disturbances. These data explain that another bias, such as osteotomy design, may be included in the comparative analysis, showing differences in the reports on the efficiency of the PE system.

## 5. Conclusions

The methodological heterogeneity among the included studies, including sample size and variability of factors, makes it difficult to draw definitive conclusions about the superiority of one method over the other in preventing neurosensory disturbances. Future studies with more robust designs, larger samples, and standardized evaluation methodologies are needed to clarify the influence of instrument type on postoperative neurosensory morbidity. However, we can conclude that the piezoelectric system offers conditions that allows a faster recovery from postoperative paresthesia.

## Figures and Tables

**Table 1:** Description of the included studies concerning the surgical procedure and its follow-up.

Authors	N	Sex F/M	Age	Surgical Technique	Surgical Movement	Complement	Method for Determining Paresthesia	Anatomical Area Affected	Follow-Up
Landes et al. [9]	50	24/26	21 ± 3	48 Le Fort I/48 MSO	ND	6 genioplasty	SLT test, sensitivity to pin-prick, TPD	Chin and lip region	3 months
Landes et al. [10]	90	56/34	26 ± 7	81 Le Fort I/94 MSO	ND	11 genioplasty	SLT test, sensitivity to pin-prick, TPD	Chin and lip region	3 months
Bertossi et al. [11]	55	ND	ND	Le Fort I/MSO	ND	ND	SLT test, sensitivity to pin-prick, TPD	Chin and lip region	12 months
Monnazzi et al. [12]	20	ND	28.4	MSO	ND	ND	Semmes–Weinstein test	Chin and lip region; the symphysis, foramen area, and vermilion	6 months
Köhnke et al. [13]	50	31/19	28.5	MSO	40 MA/10 MnR	ND	TPD/SLT	Chin and lip region	12 months
Kokuryo et al. [14]	67	50/17	ND	67 MSO	67 MnR of 8.22 ± 1.77	ND	VAS, SLT and TPD, and temperature tests	Mandibular body and chin	3 months
Sobol et al. [15]	20	8/12	19.9 ± 3.2	20 Le Fort I and MSO	ND	9 genioplasty	Functional sensory recovery scale	Lingual nerve/Mental nerve	12 months
da Costa et al. [16]	279	243/133	26 ± 11	156 Le Fort I and 376 MSO	156 MA; 147 MnA; 69 MnR	84 genioplasty	SLT to assess lingual nerve damage/TPD to assess lesion of the marginal mandibular branch of the facial nerve	Chin and lip region	36 months
Cascino et al. [17]	100	39/61	28.5	100 Le Fort I and MSO	ND	ND	VAS and standardized neurosensory tests	Chin and lip region	6 months

Obs: F: female; M: male; MSO: mandibular sagittal osteotomies; MA: maxillary advancements; MnR: mandibular retrusions; MnA: mandibular advancements; VAS: visual analog scale; TPD: Two-point discrimination; SLT: static light touch; ND: not described.

**Table 2:** Summary table of neurosensory complications from the included articles.

Authors	Postoperative Neurosensory Impairment
Landes et al. [9]	During the sagittal mandibular osteotomy procedure in male subjects with greater bone volume, longer surgical time and final sawing were required to complete the separation. Only 5% of the subjects treated with piezoelectric instruments did not recover sensitivity after 3 months, compared to 15% in the saw group. This study observed only 8% of severe fractures, possibly due to an incomplete piezo-osteotomy.
Landes et al. [10]	They do not mention postoperative complications, only reporting visual difficulty during the lingual osteotomy. Among the subjects operated on with piezoelectric instruments, 2% did not recover sensitivity after 3 months, while in the saw group, 16% did not recover sensitivity.
Bertossi et al. [11]	At 6 months, all subjects operated on with piezoelectric instruments presented with mild to moderate paresthesia, whereas 14.5% of the subjects in the saw group had no sensation. In the saw group, the inferior alveolar nerve showed a higher incidence of nerve trauma, increased bleeding, and greater cutting depth.
Monnazzi et al. [12]	All subjects presented neurosensory alterations. One month postoperatively, the areas of the lower lip, chin, and lateral aspect of the chin showed greater sensory response with the saw system. However, at two months, the aforementioned areas exhibited a greater sensory response in subjects treated with piezoelectric devices compared to those treated with a saw, although the differences were not statistically significant. These results were maintained up to six months.
Köhnke et al. [13]	All subjects presented sensory alterations in the lower lip and chin, but at six weeks, subjects operated on with the piezoelectric system showed greater sensory perception than those operated on with a saw. However, after six months, there were no sensory differences in the lower lip and chin. Age and sex variables were not associated with the degree of neurosensory alteration.
Kokuryo et al. [14]	During the first postoperative month, no neurosensory differences were observed between the two systems. However, at three months, only 22.9% of the sides in the piezoelectric group exhibited neurosensory alterations compared to 43.8% in the conventional group.
Sobol et al. [15]	Between days 94 and 123, no neurosensory differences were detected between the two systems. Variables such as age, craniofacial anomaly, and type of mandibular movement were associated with functional sensory recovery.
da Costa et al. [16]	No differences were observed when comparing total paresthesia of the lower lip and chin between 24 and 36 months postoperatively, with 16% of subjects maintaining complete loss of sensitivity in the lower lip and chin. Variables such as age and type of mandibular movement were associated with functional sensory recovery.
Cascino et al. [17]	Neurosensory differences were observed one month postoperatively, with subjects operated on using the piezoelectric system reporting fewer discomforts and requiring less medication compared to those operated on with a saw. At six months postoperatively, all subjects treated with the piezoelectric system had regained slight cutaneous sensitivity in the lower lip and chin.

## Supplementary Materials

The following supporting information can be downloaded at https://www.mdpi.com/article/10.3390/jcm14103371/s1: Table S1: Preferred reporting items for systematic reviews and meta-analyses extension for scoping reviews (PRISMA-ScR) checklist. Reference [7] is cited in Supplementary Materials.
